# Highly accurate model for prediction of lung nodule malignancy with CT scans

**DOI:** 10.1038/s41598-018-27569-w

**Published:** 2018-06-18

**Authors:** Jason L. Causey, Junyu Zhang, Shiqian Ma, Bo Jiang, Jake A. Qualls, David G. Politte, Fred Prior, Shuzhong Zhang, Xiuzhen Huang

**Affiliations:** 1 Department of Computer Science, Arkansas State University, Jonesboro, Arkansas 72467 United States of America; 2 Department of Industrial and Systems Engineering, University of Minnesota, Minneapolis, Minnesota 55455 United States of America; 3 Department of Mathematics, University of California, Davis, California, 95616 United States of America; 4 Research Center for Management Science and Data Analytics, School of Information Management and Engineering, Shanghai University of Finance and Economics, Shanghai, 200433 China; 5 Mallinckrodt Institute of Radiology, Washington University, St. Louis, Missouri 63110 United States of America; 6 Department of Biomedical Informatics, University of Arkansas for Medical Sciences, Little Rock, Arkansas 72205 United States of America; 7 The UALR/UAMS Joint Graduate Program in Bioinformatics, Little Rock, Arkansas 72204 United States of America

## Abstract

Computed tomography (CT) examinations are commonly used to predict lung nodule malignancy in patients, which are shown to improve noninvasive early diagnosis of lung cancer. It remains challenging for computational approaches to achieve performance comparable to experienced radiologists. Here we present NoduleX, a systematic approach to predict lung nodule malignancy from CT data, based on deep learning convolutional neural networks (CNN). For training and validation, we analyze >1000 lung nodules in images from the LIDC/IDRI cohort. All nodules were identified and classified by four experienced thoracic radiologists who participated in the LIDC project. NoduleX achieves high accuracy for nodule malignancy classification, with an AUC of ~0.99. This is commensurate with the analysis of the dataset by experienced radiologists. Our approach, NoduleX, provides an effective framework for highly accurate nodule malignancy prediction with the model trained on a large patient population. Our results are replicable with software available at http://bioinformatics.astate.edu/NoduleX.

## Introduction

Lung cancer is the leading cause of cancer related death worldwide^1^. Early diagnosis of lung cancer is key to reducing mortality. Screening computed tomography (CT) examinations have been shown to greatly improve noninvasive early diagnosis of lung cancer in at risk patients^2,3^. The National Lung Screening Trial (NLST) has shown that low dose computed tomography screening could reduce patient mortality by 20% compared with conventional chest radiographs^4^. The US Preventive Services Task Force (USPSTF) recommends annual screening for lung cancer with computed tomography for high risk patients. Our ability to conduct lung nodule malignancy classification from clinical CT data has important clinical impacts. However, for current clinical evaluation, radiologist’s interpretation is a tedious process, which is an impediment of clinical throughput, and highly subjective. It would be ideal to develop computational approaches, with performance comparable to radiologists, in order to aid or ease the burden of radiologists, and also potentially identify and make use of image features that may not be visually perceptible to even very experienced thoracic radiologists.

There are several substantial challenges for computational approach development with currently available CT datasets: (i) Computational approaches need to provide sensitivity to nodule features as well as robustness to noise and other artifacts introduced during data collection. (ii) Datasets need to be large enough, consistent, and ensure the integrity of expert-defined ground “truth” for approach development, training, and testing^5^. The definition of “truth” and other statistical issues regarding database design for training computer-aided diagnosis tools are discussed in^6^. The development of a successful computational approach should take these challenges into consideration.

There are two general categories of computational strategies recently developed for lung nodule malignancy prediction from CT images: (i) Radiomics approaches based on radiological quantitative image features (QIF). (ii) Deep learning approaches such as those based on convolutional neural networks (CNN). Radiomics approaches^7–14^ usually build the prediction model based on the extracted two dimensional (2D) or three dimensional (3D) radiological quantitative image features of lung nodules based on prior knowledge of what features and characteristics are significant. Radiomics approaches have been developed using publicly available datasets, such as The Lung Image Database Consortium (LIDC/IDRI) and the National Lung Screen Trial (NLST)^15^, or using proprietary datasets, which are frequently small but may be confirmed via pathology based on biopsies or surgical resections. Deep learning convolutional neural network (CNN) based approaches are very promising with the availability of CT scans from large cohorts. Many recent efforts are devoted to nodule classification approaches using convolutional neural networks^16–23^. The LIDC/IDRI cohort has been used by the authors of^16–23^ to train and test their models for classifying lung nodules.

There are several differences between the radiomics approach and the CNN based approach, which need to be taken into consideration in order to develop successful models based on either approach or potentially to integrate of the two approaches. The two approaches require different input information for nodule malignancy prediction. Radiomics approaches need proper segmentations of the nodules from radiologists or from segmentation algorithms, and then need quantitative image feature extraction. CNN approaches do not necessarily require segmentation of the nodules and can perform prediction with one marked point per nodule after the prediction model is trained. While radiomics approaches are based on radiological quantitative image features, the features learned by deep convolutional neural network approaches may be visualized as mysterious “deep dreams”^24^. Emerging as the leading machine-learning approach in the imaging domain, deep learning CNN approaches usually require a much larger training dataset, compared with radiomics approaches. Once trained, the CNN models can be more efficient for nodule malignancy prediction, compared with the models based on radiomics approaches, since the prediction can be made directly from the image without the need for a quantitative feature extraction step prior to classification.

While much progress has been made in the development of models based on either radiomics or deep learning CNNs for lung nodule malignancy classification, it remains challenging for computational approaches to achieve performance comparable to experienced radiologists. Here we present NoduleX, a novel systematic approach for lung nodule malignancy classification from clinical CT scans. The approach is based on deep learning convolutional neural network (CNN) features, and it can also integrate the information of radiological quantitative image features (QIF), if available. For training and validation with lung nodules in CT images from the LIDC/IDRI cohort, NoduleX achieves high accuracy for nodule malignancy classification, commensurate with the analysis of the dataset by experienced radiologists.

## Results

### NoduleX: an approach for nodule malignancy classification

**Figure 1:**
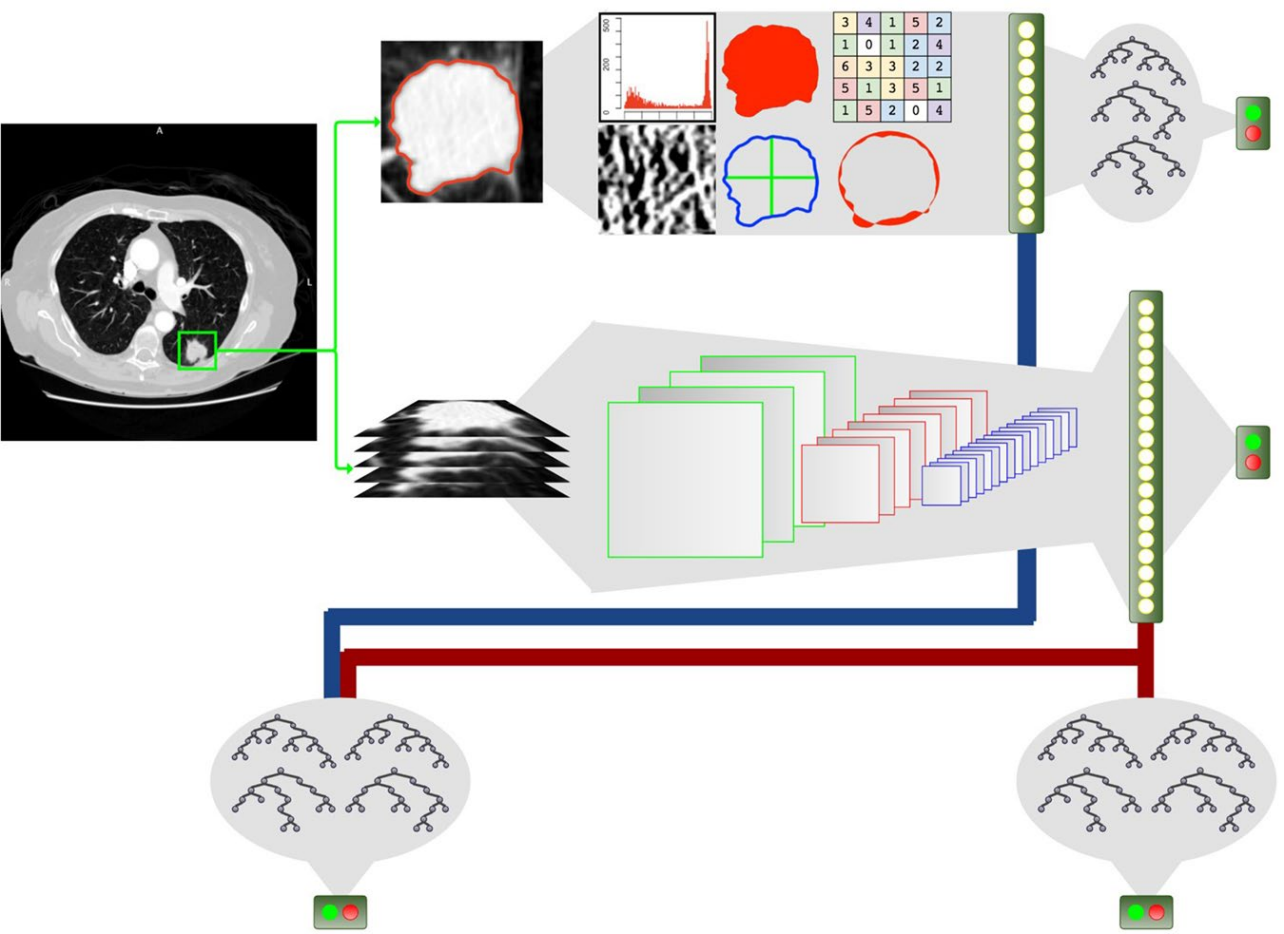
Overview of NoduleX. It takes as input one provided marked point for a region of interest, then it can generate a prediction for the classification of the nodule that matches the classification of experienced radiologists with high accuracy. If a segmentation is available, accuracy can be increased by adding quantitative image features to the model. (**a**) The prediction model based on the deep learning CNN features. A 3-D (X, Y, Z) image volume is extracted and processed through successive 2-D (X,Y) multi-channel (Z) convolutional and max pooling layers to produce spatial features that are gathered in a fully-connected layer into a 1-D “feature vector” and then to a final classification layer where a softmax function provides an output prediction. (**b**) Quantitative features extracted from the pre-segmented CT image. For our QIF model, about 50 features of the 2D and 3D images were scored for use in the combined CNN + QIF classifier. (**c**) Predictive model using the “feature vector” extracted from the CNN classifier concatenated with the features extracted from the QIF model used as input to a trained Random Forest classifier.

NoduleX is a novel, systematics approach for lung cancer nodule malignancy classification using clinical CT scans. The approach is based on deep learning convolutional neural network (CNN) features. The general framework of NoduleX is illustrated in Fig. 1. We describe the details in the Methods Section.

We followed a rigorous process for training and testing the model using CT data from the LIDC/IDRI participant cohort^15^ (see supplemental material). In the LIDC study, four experienced thoracic radiologists reviewed each of the 1018 CT cases in the LIDC/IDRI cohort and marked lesions belonging to one of three categories (“nodule > or = 3 mm,” “nodule < 3 mm,” and “non-nodule > or = 3 mm”). The lesions of nodules ≥ 3 mm have a greater probability of malignancy than lesions in the other two categories. The malignancy rating (1, 2, 3, 4, and 5) of the nodules ≥ 3 mm from the four experienced radiologists are described in detail in the two publications of the LIDC/IDRI cohort^25,26^. This malignancy score/rating of this cohort was also discussed in other related recent studies of the cohort such as^27,28^. Of the cohort, there is diagnostic data for 157 of the 1018 patients, which were obtained by performing biopsy, surgical resection, and progression or reviewing the radiological images to show 2 years of nodule state at two levels. The nodule level diagnosis of the 157 patients is: unknown, benign, malignant (primary lung cancer), and malignant (metastatic).

### NoduleX has consistent performance with high accuracy

**Figure 2:**
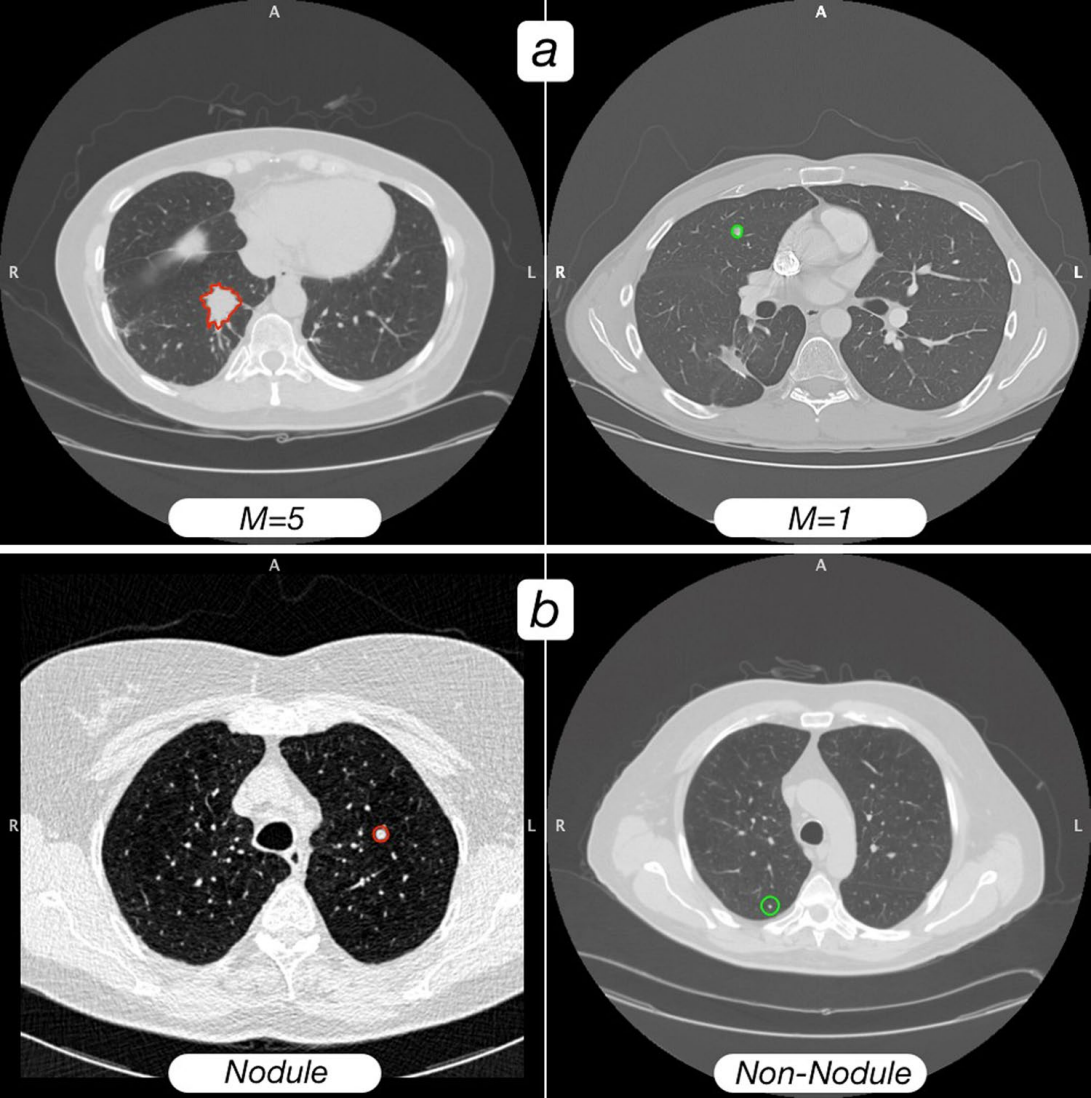
(**a**) A visual comparison of a likely malignant nodule versus a likely benign nodule. The CT scan on the left (with red ROI outline) was rated at malignancy = 5 (as highly likely malignant nodule) by consensus of the LIDC/IDRI radiologists who rated the nodule; the scan on the right (with green ROI outline) was rated as malignancy = 1 (as likely benign nodule) by consensus of the LIDC/IDRI radiologists who rated the nodule. Both outlines are from the consensus of segmentations provided by the LIDC/IDRI radiologists. (**b**) A visual comparison of a nodule versus a non-nodule. The left image is a “nodule” (rated malignancy = 3 from LIDC/IDRI) with consensus radiologist segmentation (red), and the CT scan on the right is a “non-nodule,” with computer segmentation (green). Computer segmentations were used for “non-nodules”, as LIDC/IDRI radiologists did not provide segmentations for these regions.

We processed 1065 nodules with different malignancy scores from 1–5 (with score 1 meaning highly unlikely to be malignant, score 2 or 3 indeterminate, score 4 moderately likely to be malignant, and score 5 highly likely to be malignant). The corresponding sets are denoted as S1, S2, S3, S4 and S5, respectively. We tested two designs: S1 versus S45, and S12 versus S45. For each design, the data were grouped into completely independent training and validation sets, with 80% for training and 20% for validation. Both the training and the validation sets was balanced to contain an equal number of two classes of nodules as “likely malignant” and “likely benign” nodules. Figure 2(a) shows an example of two patients’ CT scan slices of nodules with malignancy score 1 and score 5 respectively, which are reviewed by the LIDC/IDRI cohort experienced thoracic radiologists.

**Figure 3:**
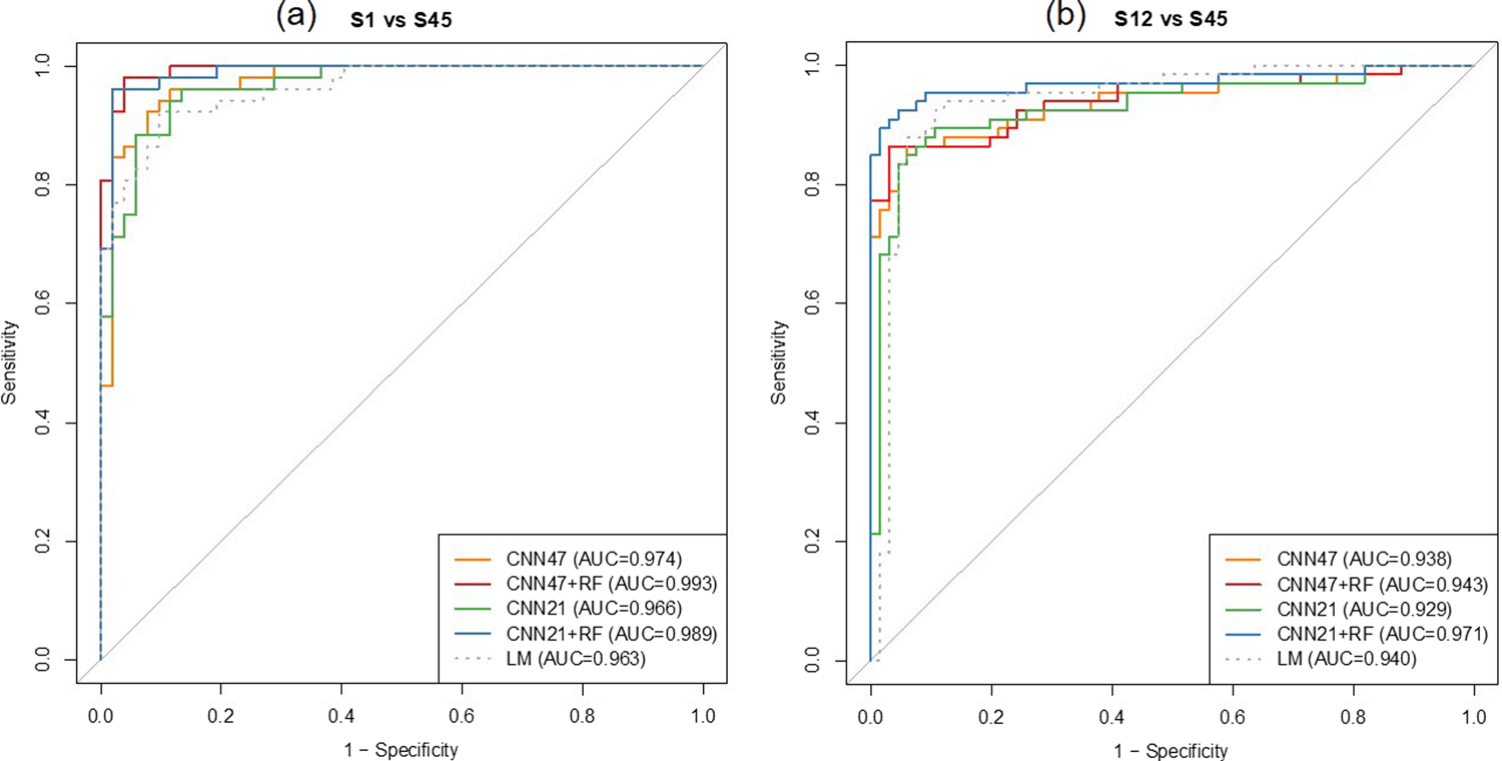
The receiver operating characteristic curves (ROC) of the NoduleX model to predict nodule malignancy rating on the validation set of two data sets: S1 vs S45 and S12 vs S45. (**a**) S1 vs S45. In this test, nodules with malignancy rating 1 were compared to nodules with malignancy ratings 4 or 5. The figure shows the comparison of two different CNN models alone, the models based on CNN features combined with QIF features, and a logistic regression model based on a measure of the nodule’s size alone as a baseline comparison. Both CNN models perform well in this task, and both are improved when QIF features are added to the model. (**b**) S12 vs S45. In this test, nodules with malignancy rating 2 were added to the “negative” class from (**a**). The figure shows the comparison of the two CNN models alone, the models based on CNN features combined with QIF features, and a logistic regression model based on a measure of the nodule’s size alone. In this test, the size metric was even more predictive, but CNN models were still competitive, and the combining QIF and CNN features again increased the overall performance of the classifier.

For the design of S1 versus S45, the best model on the validation set has an area under the receiver operating characteristic curve (AUC) of 0.974 (acc = 91.3%, sen = 88.5%, spc = 94.2%). The model performance is further improved when combined with the identified radiomic quantitative image features (QIF), with an AUC of 0.993 (acc = 95.2%, sen = 94.2%, spc = 96.2%). For the design of S12 versus S45, the best model on the validation set has an AUC of 0.938 (acc = 87.9%, sen = 87.9%, spc = 87.9%). When combined with the QIF features, the model performance is further improved with an AUC of 0.971 (acc = 93.2%, sen = 87.9%, spc = 98.5%). Figure 3(a,b) show the receiver operating characteristic curve (ROC) of two CNN models, two CNN with combined QIF features to predict malignancy on the validation set, and the baseline logistic regression model. Please refer to Table 1 for each model, the AUC, accuracy sensitivity specificity, for the validation set.

**Table 1:** Performance of NoduleX models.

Model	auc	S1 vs S45		spc	auc	S12 vs S45		spc	S0 vs S1-5		sens	spc
		acc	sens			acc	sens		auc	acc		
CNN47	0.974	0.913	0.885	0.942	0.938	0.879	**0**.**879**	0.879	0.949	0.899	0.877	0.920
CNN47 + RF	**0**.**993**	0.952	0.942	**0**.**962**	0.943	0.894	0.864	0.924	**0**.**984**	**0**.**946**	**0**.**948**	0.943
CNN21	0.966	0.913	**0**.**962**	0.865	0.929	0.886	0.864	0.909	0.945	0.880	0.835	0.925
CNN21 + RF	0.989	**0**.**962**	**0**.**962**	**0**.**962**	**0**.**971**	**0**.**932**	**0**.**879**	**0**.**985**	0.975	0.925	0.906	0.943
LM	0.963	0.885	0.865	0.904	0.940	0.826	0.697	0.955	0.689	0.538	0.358	**0**.**972**

The performance of the two CNN models (CNN47: 47 × 47 × 5 and CNN21: 21 × 21 × 5) is shown with and without the addition of QIF features (CNN47 + RF, CNN21 + RF). Each model was tested on the validation set for three datasets: S1 vs S45, S12 vs S45, and S0 vs S1-4 (“non-nodule vs nodule”). Also shown is a simple logistic regression model based on the square root of the nodule’s greatest cross-sectional area (LM) for a baseline comparison. All models are measured on area under the ROC curve (auc), accuracy (acc), sensitivity (sens), and specificity (spc). The best performance for each metric is shown in bold.

**Figure 4:**
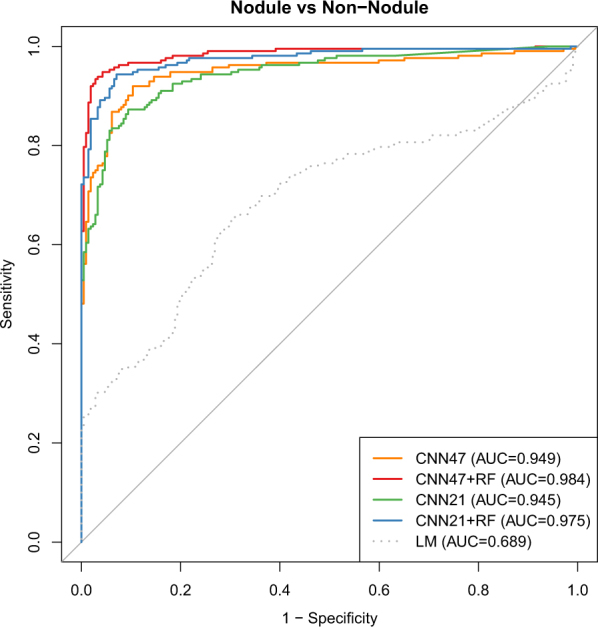
The receiver operating characteristic curves (ROC) of the NoduleX model to predict whether a region of interest is a “nodule” or “non-nodule” (S0 vs S1-5). The figure shows the comparison of two different CNN models alone, the models based on CNN features combined with QIF features, and a logistic regression model based on a measure of the nodule’s size alone as a baseline comparison. In this test, the “nodule” candidates were professionally segmented, while the “non-nodule” candidates were automatically segmented using a software package. While the separation was not as well explained by region size alone in comparison with the previous two tests, the CNN models still give an accurate classification result, and even better performance was shown when QIF features are available in combination with the CNN.

We also investigated the performance of NoduleX on prediction of nodules versus non-nodules. We used 1067 nodules with malignancy scores of 1–5 and 1056 non-nodule points, reviewed by the four radiologists of the LIDC/IDRI cohort; see Fig. 2(b) for an example of two patients’ CT scan slices of a nodule with malignancy score 3 and a non-nodule point respectively. Of the ~2000 instances of nodule and non-nodule data, 80% of the data are used for training and 20% for validation. Both the training and the validation sets were balanced to contain an equal number of nodules and non-nodule points. We tested the models’ performance on the independent validation set. For prediction of nodules versus non-nodules for the independent validation test, the model AUC is 0.949 (acc = 89.9%, sen = 87.7%, spc = 92.0%) and the model AUC is 0.984 (acc = 94.6%, sen = 94.8%, spc = 94.3%) when combined with the QIF features. Figure 4 shows the receiver operating characteristic curve (ROC) of two CNN models, two CNN with combined QIF features to predict malignancy on the validation set, and the baseline logistic regression model. Please refer to Table 1 for the AUC, accuracy sensitivity specificity, for the validation set, compared with the two designs: S1 versus S45, and S12 versus S45. In contrast to other models developed in the literature using the LIDC/IDRI dataset with AUC at the range of ~0.8, our model achieved significantly improved performance accuracy, with matched performance as experienced radiologists for the LIDC/IDRI cohort.

### Radiomics approach for nodule malignancy classification of the LIDC/IDRI cohort

Classifications (produced by a Random Forest classifier) based on the radiomics features crafted to capture visual cues that radiologists identified, in combination with radiologist segmentations, tended to agree closely with the radiologist’s assigned classifications, even when a very small number of samples were used for training. Our testing, described below and quantified in Table 2, revealed that radiomic quantitative image features are able to describe the differences in nodules that are identified by experienced radiologists as belonging to different classes (e.g., “Highly unlikely for cancer” and “Highly suspicious for cancer”). We show that even when small sample sizes are used for training, good separation can be achieved by the radiomics approach.

**Table 2:** Comparison of classification accuracy using quantitative image features (QIF) while reducing the number of examples used for fitting or training the model.

		QIF Accuracy		
		RF	RF No Size	LM
S1 vs S45	80% Train	0.962	0.962	0.885
	20% Train	0.928	0.930	0.892
	1+, 1− Train*	0.814	0.725	0.806
S12 vs S45	80% Train	0.924	0.909	0.826
	20% Train	0.865	0.874	0.868
	1+, 1− Train*	0.753	0.655	0.661
S0 vs S1-5	80% Train	0.899	0.901	0.538
	20% Train	0.865	0.871	0.588
	1+, 1− Train*	0.555	0.558	0.529

A Random Forest model including all QIF features (RF) is compared to a Random Forest model with all direct measures of size omitted (RF No Size) and a simple logistic regression model based on the square root of the greatest cross-sectional area (LM). Each model is fit with 80% of the available data, reserving 20% for testing, then again by reversing the train/test ratio (20% for training, 80% for testing). As an extreme comparison, the models are trained/fitted with a single example from each class (1 positive and 1 negative example) for training, then tested against the remaining examples. ^*^The 1+, 1− training process was repeated 200 times, choosing the two training examples at random each time; the accuracy score shown is the average score over all 200 trials.

To establish a baseline for the separation difficulty, a logistic regression model (LM) was trained only on a size metric (square root of largest cross-sectional area). This metric was chosen as an analogue to the RECIST metric^29^, in which the longest cross-sectional diameter is measured. For S0 vs S1-5, i.e., non-nodule vs nodule, the separation test was also conducted. The LM baseline is shown in Figs 3 and 4, and in Table 2.

To establish a lower limit on how well the radiomics features, with a Random Forest (RF) model, could classify the nodules, we conducted the following tests:

S1 vs S45 RF separation using 1+ and 1− training set:

1 positive (S45) nodule and 1 negative (S1) nodule chosen at random from the full set of 520 nodules were used for training a RF classifier, with the remaining 518 as the “test set”; the test was repeated 200 times, and the results averaged: mean AUC = 0.91; mean acc = 81%.

S12 vs S45 RF separation using 1 + and 1− training set:

1 positive (S45) nodule and 1 negative (S12) nodule chosen at random from the full set of 664 nodules were used for training a RF classifier, with the remaining 662 as the “test set”; the test was repeated 200 times, and the results averaged: mean AUC = 0.86; mean acc = 75%.

Additionally, to establish a baseline for the separation difficulty, a logistic regression model (LM) was trained only on a size metric (square root of largest cross-sectional area). For S0 vs S1-5, i.e., non-nodule vs nodule, the separation test was also conducted.

Please refer to Table 2 for the comparison of the quantitative image feature (QIF) models with the baseline model for the designs of S1 vs S45, S12 vs S45, and S0 vs S1-5.

## Discussion

**Figure 5:**
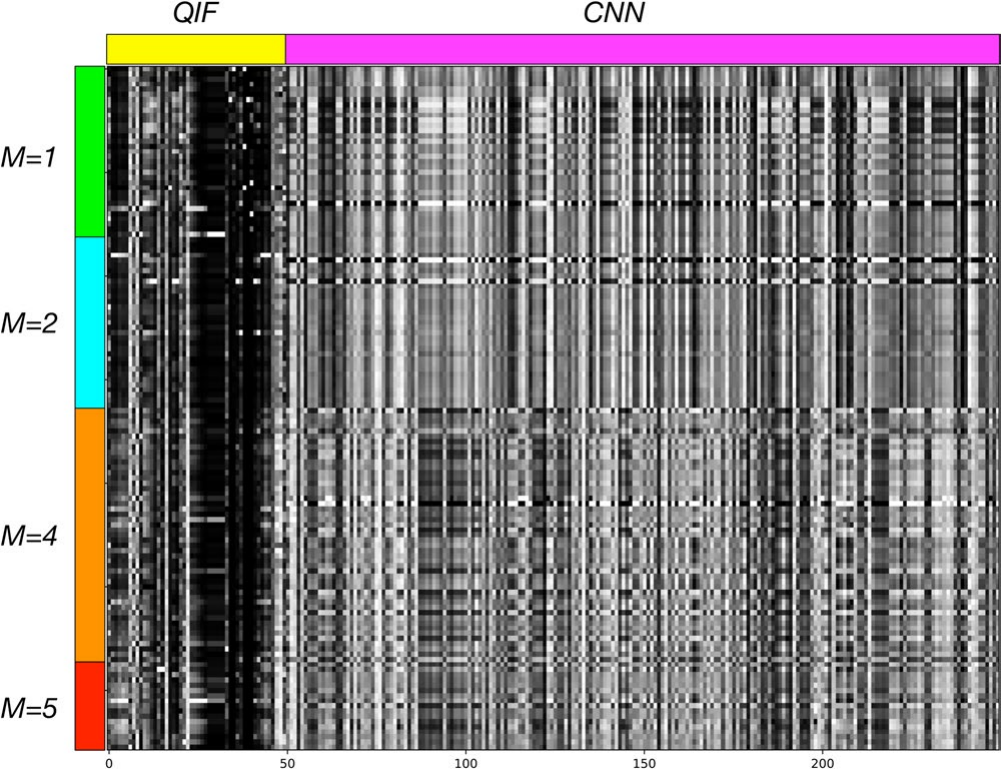
Visual illustration of 250 feature values (in columns) from both QIF and CNN features for each nodule (in rows) in the “S12 vs S45” experiment. Row groups containing M = 1, 2, 4, 5 nodules are marked, as is the transition between columns containing the 50 QIF and 200 CNN features. The CNN was trained to differentiate the M = 1,2 nodules from M = 4,5 nodules; a visible difference in features can be seen at the transition point. Differences within each class are more subtle.

We present NoduleX, an effective framework for lung nodule malignancy prediction from patients’ CT scans. For training and validation, we performed analysis of the nodules of the LIDC/IDRI cohort and we found that NoduleX can achieve ~0.99 AUC on the independent validation test. Unlike existing models usually with moderate accuracy levels, our testing results demonstrate that NoduleX can achieve high prediction accuracy, commensurate with the reviews of the cohort from experienced radiologists. The NoduleX model was developed with a deep CNN architecture, capable of performing classification or producing a feature vector that can be used as input to a secondary classifier such as Random Forest, XGBoost or AdaBoosting. The training and validation sets were carefully designed from the LIDC/IDRI cohort to avoid potential statistical biases or issues. The model is trained using the nodules with different malignancy score of the LIDC/IDRI cohort and validated using completely independent validation sets developed from the cohort to avoid patient overlap between the training and validation sets. (iii) The model can integrate deep learning CNN features (CNN feature expression) with radiological quantitative image features (radiomic expression) if a segmentation of the nodules is available. The CNN and the QIF models produce features through very different processes but produce consistent classification performance. Combining these two types of features can improve performance further. Figure 5 shows a graphical representation of the features from the S12 versus S45 experiment. In the figure, rows represent nodules and columns are individual features. The first 50 features are from the QIF data, the remaining 200 are from the feature layer of the CNN (CNN47 features are shown). The figure is labeled to indicate the consensus malignancy rating of each nodule from the LIDC radiologists. Features (columns) were scaled linearly the range [0 - 255] to facilitate the visualization.

There are in general three ways of using the data of the LIDC/IDRI cohort for the study related to classifying nodule as two classes of “likely benign” and “likely malignant”:

(i) Using the set of nodules with the malignancy score/ratings (1, 2, 3, 4, and 5), reviewed by the experienced radiologists of the cohort. We treat the malignancy of nodules as a binary classification problem for “likely malignant” versus “likely benign” by thresholding the radiologist-assigned malignancy values so that malignancy values below 3 (i.e., 1 and 2) are categorized as benign and values above 3 (i.e., 4 and 5) are categorized as malignant. Recent published models for the problem of classifying S12 (considered as “likely benign”) versus the S45 (considered as “likely malignant”) include the models developed in^20^ and^28,30–32^. In^20^, a multi-scale CNN (MCNN) approach is used to produce a feature vector of size 50 that is then input to a random forest classifier. The method achieved accuracy of 86.84%. In^30^, Taxonomic indexes and phylogenetic trees were used as texture descriptors, and a Support Vector Machine was used for classification. The proposed method shows promising results for accurate diagnosis of benign and malignant lung tumors, achieving an accuracy of 88.44%, sensitivity of 84.22%, specificity of 90.06% and area under the ROC curve of 0.8714.
(ii) Using only the set of nodules of the patients with diagnosis data. As in^33^, only those nodules with benign and malignant labels are considered. They used 52 subjects with malignant nodules and 21 subjects with benign nodules with a total of 458 and 107 individual lung nodules respectively. The obtained results are very encouraging, correctly classifying 82% of malignant and 93% of benign nodules on unseen test data at best. Similarly in^34^ the authors used the clinically provided pathologically-proven data as ground truth, and obtained an average accuracy of 77.52% with a sensitivity of 79.06% and specificity of 76.11%.
(iii) Using both sets. The authors in^18^ used the ratings from diagnostic data as the ground truth for training the classification system and evaluating the results instead of using the radiologists provided ratings. In^18^, the author reported the trained model obtained an overall accuracy of 75% for classifying whether the nodule is benign or malignant.

Our rationale for the Nodule versus Non-Nodule classification task was to use the full LIDC/IDRI cohort to address the task first presented in the LUNA16 grand challenge of *LUng Nodule Analysi*s^35^.

There are several common pitfalls when evaluating and testing a classification model for potential lung nodule malignancy: (i) not using a completely independent validation set; (ii) not considering the nodules of the same patient to be completely separated into the training set or the validation set; (iii) not testing the model without size-related image features in order to remove the potential bias introduced by nodule size; (iv) not reporting the classification testing results with the complete information for AUC, as well as for accuracy, sensitivity, and specificity. For example, if an unbalanced number of “likely benign” and “likely malignant” nodules for the validation set is used, the testing result may have a very high accuracy rate but with a very low specificity.

As is pointed out in^33^, since most of the research groups report experimental results using their own proprietary dataset that is not publicly available or a different subset of a publicly available dataset, a direct absolute comparison of their statistics performance is not possible. Therefore, it is difficult to cross-validate the developed computational approaches with a completely different dataset. Please refer to a summary of related works in “likely benign” vs “likely malignant” lung nodule classification in^33^.

Evaluating the performance of NoduleX introduces several challenges: (i) The number of patient CT scans in the LIDC/IDRI cohort is not large enough for training very sophisticated CNN classification models. For example, with a large number of images, the learning model for face recognition of Facebook was trained on four million images and it is said that the model reached an accuracy level even higher than the FBI current system for face recognition. Other groups, including^20–23^ have trained CNN models with fewer layers; this would help alleviate the training difficulty by reducing the total parameter count. As pointed out in^22^, it is possible that deeper CNNs may not offer much improvement over more shallow models. (ii) We performed single-partition evaluation, where our validation set was carefully chosen so that no patient had nodules appearing in both the training and validation partitions. We chose this approach in alternative to N-fold cross-validation, since it is likely that some patients would have nodules included in two or more partitions in such an approach. However, this also means that our results may not be as statistically robust as those obtained through cross-validation. We chose a tradeoff to reduce a potential information-leakage bias, but acknowledge that this approach is susceptible to other biases. For comparison^20–23^, performed experiments on the LIDC/IDRI dataset with CNN models and chose to use cross-validation. (iii) For training and validating we use the LIDC/IDRI dataset. It may not be directly applicable to other datasets with very different CT scan image quality or different definitions for ground truth classifications. If two datasets are of very different quality or if nodule ground truth labels are not consistent, the computational model trained on one dataset will need to be re-trained in order to work for the other dataset. We may consider for future research work on transfer learning and testing on other screening and diagnostic data of CT images. There are some recent works on transfer learning for survival prediction analysis such as in^36^. (iv) We cannot make a direct performance comparison of our model with previously developed models. Although the models developed in^20,31^ were trained and tested on the same problem of classifying S12 vs S45, the nodule sets were chosen differently from the LIDC/IDRI cohort. A standardized dataset would helpful in this regard, similar to the way well-known datasets like ImageNet have helped comparisons in natural image recognition. (v) The nodules of the LIDC/IDRI cohort with malignancy scores of 4 or 5 were not necessarily confirmed through biopsies or surgical resection as real malignant nodules. We plan to test and cross-validate the approach with other datasets where diagnostic truth has been established.

Interestingly, our testing also reveals the problem of lung nodule malignancy prediction with the available nodule classification from experienced radiologists as ground truth of the LIDC/IDRI cohort, which has been widely studied in the area, is actually a relatively “easy” radiomics problem. Our testing shows that radiomics features are able to describe the differences in nodules that are identified by a human expert as belonging to very different classes. Our testing results demonstrate that even when small sample sizes are used for training, a radiomics prediction model can achieve reasonably good separation. This lends confidence that the radiomic quantitative features are robustly representing information that a human would use to classify the nodules. Of course, it leaves open the question of whether these nodule-level classifications are a good predictor of patient outcome – additional research on a dataset where outcome is included will be necessary to address this.

With NoduleX as a systematic framework, the model can be re-trained with other large datasets of CT scans, and we anticipate it can achieve similar high accuracy on other datasets. It can be re-trained when new data becomes available and can continue to learn from the increasing knowledge of radiologists as well as be further trained with more available CT scans from a larger population. This research will help open a new path for developing effective computational approaches based on deep CNN features in medical imaging, which may have a clinical impact as images are routinely collected in clinical practice, for disease diagnosis, prognosis and treatment.

The CNN component of NoduleX (and similar CNN-based models such as^16–23^) promises an advantage over quantitative feature models in that no detailed segmentation is required. These segmentations require either tedious work on behalf of a trained radiologist, or error-prone automated segmentation algorithms. We saw in this work that when quality segmentations are available, QIF features perform very well alone, and may be added to CNN features to further improve classification. In our “Nodule versus Non-Nodule” experiment, we had to use an automated segmentation algorithm, as no segmentations were provided from LIDC/IDRI. This technique tended to produce segmentations that were larger and more “circular” than the visible region of interest in the image. Despite this inaccuracy, the QIF model still performed well for the task, and still improved accuracy when added to the CNN features. It can be seen in Fig. 4 that the baseline (LM) model did not perform well at this task, likely due to the tendency of the algorithm to create inaccurate segmentations whose area was too large.

NoduleX makes a substantial step towards addressing the challenge that computational prediction for lung nodule malignancy with patients CT scans can have matched performance as the reviews of experienced thoracic radiologists in current clinical practice. We performed analysis of >1000 nodules of the CT scans of 1018 patients of the LIDC/IDRI cohort. All these nodules were identified and classified by four experienced thoracic radiologists who participated in the LIDC project. Our test shows that NoduleX, when provided with CT data and a point locating the nodule, achieves an area under the receiver operating characteristic curve (AUC) of 0.97. The performance is further improved when combined with quantitative image features (QIF), resulting in an AUC of 0.99. Compared with previous work for this problem based on QIF or CNN in the literature, NoduleX achieved significant performance accuracy, commensurate with the reviews of the LIDC/IDRI cohort by the experienced radiologists. As the recent great advancements in deep learning approaches for voice recognition and face recognition with large available datasets, we expect advancements in computational learning approaches in biomedical imaging for lung cancer early detection and diagnosis. Valuable future work would include experiments to determine ideal model architectures for extracting various kinds of features from radiological images, some of which has been begun by^22^. Additionally, we hope that curated train/test datasets may be produced for radiomics research in the same way they have been created for natural image machine learning research.

## Methods

The methods were carried out in accordance with relevant guidelines. The authors acknowledge the National Cancer Institute and the Foundation for the National Institutes of Health, and their critical role in the creation of the free publicly available LIDC/IDRI Database^25,26^ used in this study. This dataset is freely available to browse, download, and use for commercial, scientific and educational purposes as outlined in the Creative Commons Attribution 3.0 Unported License.

### NoduleX for nodule malignancy classification

**Figure 6:**
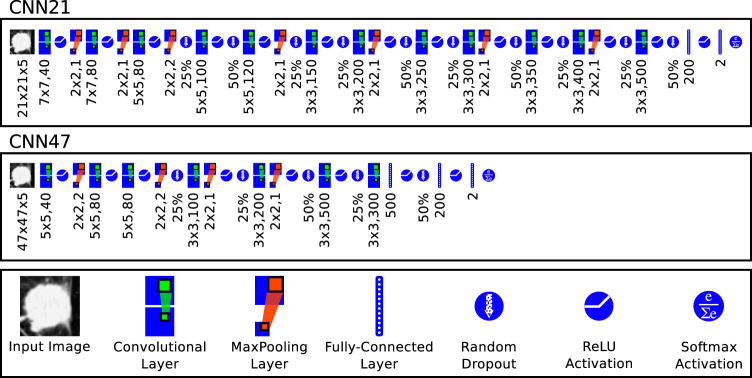
The layout of the two CNN networks. CNN21 is the network whose input size is 47px. × 47px. × 5 slices; CNN47 is the network whose input size is 47px. × 47px. × 5 slices. Both networks produce a final classification probability for two classes. We used the same network layout for the S1vS45, S12vS45, and Nodule VS Non-Nodule classifiers, although we trained separate models for each. The legend (bottom box) defines symbols used to represent each major component of the network. The numbers below each symbol in the layout graphs refer to the parameter settings at each stage. For convolution layers, $x\times y,{n}_{f}$ represent the width $x$ and height $y$ of the filter and ${n}_{f}$ represents the number of filters learned at that stage. For max-pooling layers, *x* × *y*, *s* represent the width $x$ and height $y$ and stride $s$ (symmetric in both x- and y-axes). The percentage shown for dropout layers indicates the percent of units that are randomly dropped. The number shown for fully-connected layers indicates the number of units in that layer.

Here we describe the details of NoduleX, for lung nodule malignancy classification using clinical CT scans. The approach is based on deep learning convolutional neural network (CNN) features. The general framework of NoduleX is illustrated in Fig. 1 and the CNN network layout for the two networks reported here is illustrated in Fig. 6.

### Nodule Selection

The nodule annotations provided in XML format with the LIDC/IDRI CT image dataset were used to produce a consensus list of nodules for each patient such that the nodules in the consensus list had no overlap and the malignancy rating assigned was the average of the malignancy ratings assigned by the radiologists who annotated the nodule, rounded to the nearest integer. One nodule was then chosen at random from each patient in the study who had at least one lesion marked as a “nodule ≥ 3 mm” by at least one radiologist. This random selection produced 44 nodules rated “malignancy = 1” and 270 nodules rated “malignancy = 4” or “malignancy = 5”; to balance the size of the positive and negative classes, an additional 206 nodules rated “malignancy = 1” were selected by choosing all unique nodules rated “malignancy = 1” from patients for whom a nodule rated “malignancy = 4” or “malignancy = 5” was not selected in the random selection phase. (Note: some of these patients do have “malignancy = 4 or 5” that were not selected by the initial random sampling procedure.) This resulted 250 nodules rated “malignancy = 1” and 270 nodules rated “malignancy = 4” or “malignancy = 5”. Table 3 shows the upper bound and an estimate for the number of ratings received by each nodule selected for any of our experiments. While all nodules in LIDC/IDRI were rated by at most 4 radiologists, our consensus method can produce higher numbers of ratings per nodule if a nodule had complex structure such as inclusions or “lobes” that appear as separate segmented regions in some slices, or if the consensus nodule was actually two or more nodules from the original dataset that merged as a result of overlapping voxels in the segmentation mask. Thus, we note the upper bound on the number of ratings by examining the slice with the maximum number of ratings; if this number is ≤4, we know that the total number of raters must be ≤ this number. If this number is >4, we report it as 4 since we know no more than 4 radiologists rated any single nodule. For a more realistic estimate of the number of raters, we look at the number of ratings received most often by all the slices in the nodule (similar to the “mode” of the number of ratings per slice), taking the larger value in case of ties. Additional statistics information for the consensus nodules is provided as a supplementary spreadsheet.

**Table 3:** Listing of the number of nodules rated by at most 1,2,3, or 4 radiologists, and the number of nodules according to an estimated number of raters.

	All		M1		M2		M3		M4		M5	
Rated by at most 1	506	0.277	117	0.391	258	0.385	83	0.204	36	0.101	12	0.129
Rated by at most 2	309	0.169	62	0.207	149	0.222	59	0.145	35	0.098	4	0.043
Rated by at most 3	299	0.164	34	0.114	116	0.173	79	0.194	57	0.160	13	0.140
Rated by at most 4	713	0.390	86	0.288	148	0.221	186	0.457	229	0.641	64	0.688
Est. to be rated by 1	543	0.297	121	0.405	267	0.398	91	0.224	46	0.129	18	0.194
Est. to be rated by 2	324	0.177	60	0.201	154	0.230	57	0.140	42	0.118	11	0.118
Est. to be rated by 3	300	0.164	34	0.114	107	0.159	83	0.204	60	0.168	16	0.172
Est. to be rated by 4	660	0.361	84	0.281	143	0.213	176	0.432	209	0.585	48	0.516

Counts are shown first for all nodules, and for nodules of each malignancy consensus class 1–5, with ratios shown to the right of each count. Estimated number of ratings was determined by counting the number of ratings that were recorded on the largest number of slices (similar to a “mode” of the number of ratings per slice). In case of ties, the larger number was chosen.

### CNN Input Volume Extraction

Input to the CNN consists of a small 3-D volume measuring either 21 pixels × 2 pixels × 5 slices, or 47 pixels × 47 pixels × 5 slices, depending on the CNN architecture. These volumes were extracted from the full CT scan by selecting the 3D region centered around the nodule’s center of mass (centroid), as determined by consensus of the segmentations from all radiologists who segmented the nodule (the average centroid among all segmentations was used). These rectangular volumes were saved along with the consensus malignancy rating of the nodule, the minimum and maximum pixel intensities of the scan, and a class identifier in a file in HDF5 format. Separate “train” and “validation” sets were created as separate HDF5 files in this way; the “train” set contained 442 nodules and the “validation” set contained 110 nodules; all nodules in the “train” and “validation” sets were matched to corresponding nodules in the QIF “train” and “validation” sets.

### CNN Training

The CNN was trained by further dividing the “train” set into a training group consisting of 80% of the included nodules and a testing group containing the other 20%; this division was performed at random at the start of each training run. Training continued for 200–400 epochs, and the batch size was 64. At the end of each epoch a checkpoint of the model weights was saved if the model loss was improved. The final model weights as well as the three checkpoints with the highest accuracy on the testing portion of training data were retained. To reduce overfitting, automatic data augmentation was performed in which each input image volume was randomly shifted up to 30% in both the X and Y directions, and randomly rotated between 0 and 180 degrees. After training, the retained model weights were evaluated against the separate “validation” set; results for the best 21 × 21 × 5 and 47 × 47 × 5 model are reported. The *Keras* software package with the *Theano* backend was used for implementation, training, and testing of the CNN model.

### CNN Feature Extraction

After training is completed, features are extracted from the validation set by providing the set of nodule volumes to be evaluated to the CNN network in prediction mode. Output values are captured from the fully-connected layer just prior to the 2-class classification layer (i.e. the second-to-last layer). These values form a feature vector for each nodule, and are aggregated into an output CSV file for further processing in combination with QIF features.

### NoduleX Classification

Nodule classification was performed using two different CNN models (the CNN21 and CNN47 models) and both with and without QIF features. For classification without QIF features, the CNN model’s own output softmax classifier was used for class prediction on all nodules in the validation set. When QIF features are used, a vector representing the 50 QIF features is concatenated with the feature vector produced by the CNN as described above, producing a feature vector with 250 features. This combined feature vector is passed as input to a Random Forest classifier model, which itself must be trained on the training set (as described above). The trained Random Forest is then evaluated on the same validation set as the CNN-only model for comparison. The *randomForest* package^37^ in R was used for this purpose, with the *ntrees* parameter set to 1000 and defaults for other parameters.

### Description of the datasets

The LIDC/IDRI datasets contains the CT scans of 1018 patients/cases, and some patients may have more than one nodule. These CT scans were reviewed by four experienced thoracic radiologists. The radiologists annotated each scan by marking regions of interest in three classes: “nodule ≥ 3 mm,” “nodule < 3 mm,” and “non-nodule.” Each nodule in the “nodule ≥ 3 mm” class was then given a malignancy score and a detailed segmentation. “Non-nodule” and “nodule < 3 mm” regions were noted by position in the scan only. The malignancy scores were defined as follows: 1 “Highly Unlikely for Cancer,” 2 “Moderately Unlikely for Cancer,” 3 “Indeterminate Likelihood,” 4 “Moderately Suspicious for Cancer,” 5 “Highly Suspicious for Cancer.”

### Dataset S1 vs S45

This dataset consists of 520 nodules with malignancy ratings of 1, 4, or 5 as determined by consensus of radiologist ratings from the metadata provided with the LIDC/IDRI cohort. Nodules with malignancy = 1 were designated as the “negative” class (S1) and nodules with malignancy = 4 or 5 were designated as the “positive” class (S45). There were 250 S1 nodules and 270 S45 nodules in total.

### Dataset S12 vs S45

This dataset consists of 664 nodules with malignancy ratings of 1, 2, 4, or 5 as determined by consensus of LIDC/IDRI radiologist ratings. Nodules with malignancy = 1 or 2 were designated as the “negative” (S12) class, and nodules with malignancy = 4 or 5 were designated as the “positive” (S45) class. There were 394 S12 and 270 S45 nodules in total.

### Dataset S0 vs S1-5

This dataset consists of 1056 regions classified as “non-nodules” and 1069 regions classified as “nodules” by the LIDC/IDRI radiologists, with different malignancy scores (specifically with about 251, 143, 405, 204, and 66 of nodules assessed with a malignancy score of 1, 2, 3, 4, and 5, respectively).

### Description of the models of NoduleX

NoduleX is based on the deep convolutional neural network (CNN) feature expression as well as the radiological quantitative image feature expression. For predicting malignant lung nodules using CT scan images, we trained and validated the three kinds of models: the QIF model based on the radiological quantitative image features (abbreviation: QIF model), the CNN model based on deep convolutional neural networks (abbreviation: CNN model), and the combined model based on the QIF and CNN features (abbreviation: QIF + CNN model). We used completely separated datasets for training and for validation. We conducted a systematic study and comparison of the models.

### The process for training the deep learning convolutional neural networks (CNN)

For the CNN models we used the centroids of our consensus segmentation for all “nodule ≥ 3 mm” regions, or location provided by the radiologists for the “non-nodule” regions. We extracted a 3-D region of the CT image centered around this location for input into the CNN. The size of this image region varied according to the CNN model. We tested models with input (X × Y × Z) sizes 47 × 47 × 5; 21 × 21 × 5; 21 × 21 × 3; 31 × 31 × 3. We trained both 2-D multi-channel CNNs using one “slice” (Z-axis) as one “channel” of the input image, as well as 2.5-D CNNs using 3 orthogonal (along each axis) slices, and 3-D CNNs. We achieved the best tradeoff in training robustness and time by using the 2-D multi-channel approach, which is presented here. We used data augmentation during training to offset the relatively small number of examples available in the dataset; each input image was randomly shifted, scaled, and rotated by varying amounts to produce a larger effective training set. The model was trained for a specific number of epochs (200–400) and a snapshot of the model weights was taken each time a new minimum testing loss was achieved during the training process. The final model weights as well as the three snapshots with the lowest loss were retained for validation.

### The process of Radiological quantitative image (QIF) features extraction

Radiological quantitative image features analysis of the nodules reviewed by radiologists of the LIDC/IDRI cohort was performed with a similar process as detailed in^9^. Segmentations for all “nodule ≥ 3 mm” regions were provided by the LIDC/IDRI study; we used a consensus method to combine the multiple segmentations and malignancy ratings provided for each nodule. Consensus segmentations were obtained by plotting each of the radiologist provided segmentations (1 to 4 per nodule per slice); any voxel included in ≥50% of available segmentations was included in the consensus segmentation. The consensus malignancy rating was the average of all malignancy ratings assigned to all slices included in the final consensus segmentation, rounded to the nearest integer. “Non-nodule” regions were segmented using an automated Python software library. The segmented regions were further processed by a Matlab/Octave library to produce the quantitative image feature measurements. Our quantitative image features were chosen based on the work of^9^. We used the following 50 2-D features: Transverse plane with maximal area, Number of pixels inside nodule, Area, Span of nodule (in mm.) in both x and y directions, Perimeter, Circularity, Primary and secondary rotational moments, Ratio of largest to smallest rotational moment, Median of HU inside nodule, Mean of HU inside nodule, Standard Deviation of HU inside nodule, Variance of HU inside nodule, Skewness of HU inside nodule, Kurtosis of HU inside nodule, Entropy of HU inside nodule, Mean of difference image of HU inside nodule, Standard Deviation of difference image of HU inside nodule, Variance of difference image of HU inside nodule, Skewness of difference image of HU inside nodule, Kurtosis of difference image of HU inside nodule, Entropy of difference image of HU inside nodule, Lacunarity at 10 box sizes (${2}^{i},{i}=\mathrm{1,2},\ldots ,10$), Coarseness at distances of 1 and 2 pixels, Contrast at distances of 1 and 2 pixels, Busyness at distances of 1 and 2 pixels, Complexity at distances of 1 and 2 pixels, Texture Strength at distances of 1 and 2 pixels, Summed distance to surface, Mean distance to surface, Normalized summed distance, Normalized mean distance, Fractal dimension of area, Fractal dimension of perimeter, and Gradient margin. A detailed discussion of the features, their interpretation, and their relative importance in nodule classification is presented in^9^.

### Software Availability

Our results are replicable with software available at http://bioinformatics.astate.edu/NoduleX.

## Electronic supplementary material

Supplementary Information
